# Mammalian MutY Homolog (MYH or MUTYH) is Critical for Telomere Integrity under Oxidative Stress

**DOI:** 10.21926/obm.geriatr.2202196

**Published:** 2022-04-02

**Authors:** Aditi Gupta, Bor-Jang Hwang, Daniel Benyamien-Roufaeil, Sara Jain, Sophie Liu, Rex Gonzales, Robert A. Brown, Michal Zalzman, A-Lien Lu

**Affiliations:** 1. University of Maryland School of Medicine, Baltimore, MD, USA; 2. University of Maryland School of Medicine; The Center for Stem Cell Biology and Regenerative Medicine; Marlene and Stewart Greenbaum Cancer Center, Baltimore, MD 21201, USA; 3. University of Maryland School of Medicine; Marlene and Stewart Greenbaum Cancer Center, Baltimore, MD, USA

**Keywords:** MutY homolog (MYH or MUTYH), SIRT6, Rad9/Rad1/Hus1 clamp, telomeres, oxidative stress, DNA damage response

## Abstract

Telomeres consist of special features and proteins to protect the ends of each chromosome from deterioration and fusion. The telomeric DNA repeats are highly susceptible to oxidative damage that can accelerate telomere shortening and affect telomere integrity. Several DNA repair factors including MYH/MUTYH DNA glycosylase, its interacting partners Rad9/Rad1/Hus1 checkpoint clamp, and SIRT6 aging regulator, are associated with the telomeres. MYH prevents C:G to A:T mutation by removing adenine mispaired with a frequent oxidative DNA lesion, 8-oxoguanine. Here, we show that *hMYH* knockout (KO) human HEK-293T cells are more sensitive to H_2_O_2_ treatment, have higher levels of DNA strand breaks and shorter telomeres than the control *hMYH*^+/+^ cells. SIRT6 foci increase at both the global genome and at telomeric regions in H_2_O_2_-treated *hMYH*^+/+^ cells. However, in untreated *hMYH* KO HEK-293T cells, SIRT6 foci only increase at the global genome, but not at the telomeric regions. In addition, the *hMYH* KO HEK-293T cells have increased extra-chromosomal and intra-chromosomal telomeres compared to the control cells, even in the absence of H_2_O_2_ treatment. After H_2_O_2_ treatment, the frequency of extra-chromosomal telomeres increased in control HEK-293T cells. Remarkably, in H_2_O_2_-treated *hMYH* KO cells, the frequencies of extra-chromosomal telomeres, intra-chromosomal telomeres, and telomere fusions are further increased. We further found that the sensitivity to H_2_O_2_ and shortened telomeres of *hMYH* KO cells, are restored by expressing wild-type hMYH, and partially rescued by expressing hMYH^Q324H^ mutant (defective in Hus1 interaction only), but not by expressing hMYH^V315A^ mutant (defective in both SIRT6 and Hus1 interactions). Thus, MYH interactions with SIRT6 and Hus1 are critical for maintaining cell viability and telomeric stability. Therefore, the failure to coordinate 8-oxoG repair is detrimental to telomere integrity.

## 1. Introduction

Reactive oxygen species (ROS) are produced as byproducts of endogenous cellular metabolism, or through exposure to external agents, and are major threats to genomic integrity [1]. Oxidative DNA damage has been implicated in aging, neurodegenerative diseases, and cancer [2]. Particularly, the mammalian telomeric DNA (TTAGGG) repeats are highly susceptible to oxidative damage [3-7]. Guanine is frequently oxidized to highly mutagenic lesion 8-oxo-7,8-dihydroguanine (8-oxoG, G°) [8]. If not repaired, G° mispairs with adenine during DNA replication resulting in a G:C to T:A mutation [9-11]. Oxidative DNA lesions are primarily repaired by the base excision repair (BER) pathway [12-14]. In mammalian cells, the misincorporated adenines in A/G° mismatches are removed by the MutY homolog (MYH or MUTYH)-directed BER pathway [9, 15, 16]. Individuals with germline mutations in the human *MYH* (*hMYH*) gene are susceptible to colorectal cancer as well as other cancers (as in MYH-associated polyposis or MAP) [17].

Telomeres, located at the ends of chromosomes, consist of special features and proteins to prevent chromosome deterioration, unnecessary recombination, and fusion [18]. Telomere instability is linked to germline and somatic degenerative diseases as well as cancer [19]. Oxidative damage to telomeric DNA causes telomere attrition over time [6, 7] and triggers cellular senescence [20, 21]. It has been shown that persistent G° at telomeres promotes telomere shortening, aberration, and crisis [22, 23]. Therefore, telomeres are reliant upon efficient DNA repair to maintain their integrity [4, 5, 24]. Several DNA repair factors are associated with telomeres [13] and interact with telomere binding proteins [25, 26]. We have shown that hMYH DNA glycosylase is associated with telomeres and that mouse Myh (mMyh) foci are induced on telomeres by oxidative stress [25, 26]. Factors that stimulate base excision repair (BER) processes including Rad9/Rad1/hus1 (9-1-1) checkpoint clamp and aging regulator SIRT6 protein deacetylase, which are also associated with telomeres, are essential for telomere genomic stability [26-30]. We have shown that MYH, SIRT6, and 9-1-1 form a complex to maintain genomic stability in mammalian cells [25]. These results highlight the importance of the roles of BER in telomere maintenance. To examine the role of hMYH on telomere stability, we have knocked out *hMYH* gene in human HEK-293T cells and examined their cellular response to oxidative stress and alteration of telomere phenotypes. We show that hMYH is critical for maintaining cell viability and telomeric integrity under oxidative stress.

## 2. Materials and Methods

### 2.1 Cell Culture

HEK-293T and *hMYH* knockout HEK-293T cells have been described [26]. The cells were maintained in DMEM (Corning Cellgro) supplemented with 4.5 mg/ml D-glucose, 2 mM L-glutamine, 1 mM sodium pyruvate, 1X Penicillin/Streptomycin and 10% fetal bovine serum.

### 2.2 Transfections and Peroxide Treatment

Plasmids *pEGFP-hMYH* [26], pEGFP-*hMYH*^*V315A*^ [26], pEGFP-*hMYH*^*Q324H*^ [26], and pEGFP-C1 vector (Clonetech Laboratories) were transfected into *hMYH* knockout HEK-293T cells with X-tremeGENE™ HP DNA Transfection Reagent (Millipore Sigma), according to the manufacturer’s protocol. The cells were replated 48 hours after the transfection, and stably transfected cells were selected with 75 μg/ml Geneticin (G418). Transfection was confirmed by green fluorescence under DMi8b fluorescent microscope (Leica). For peroxide treatment, cells were seeded on culture plates for one day and then treated with 150 μM H_2_O_2_ for 1 hour, then recovered in fresh medium between 2 hours to 10 days or remained untreated as controls.

### 2.3 Colony Formation Analysis

Cells were treated with 150 μM H_2_O_2_ for 1 hour or left untreated, followed by incubation for 10 days. Colony formation was analyzed as described [27].

### 2.4 Immunofluorescence Staining

Stably transfected *hMYH* KO HEK-293T cells were treated or remained untreated with H_2_O_2_ as described above, fixed, permeabilized, and reacted with primary antibody against phosphorylated H2AX (γH2AX) (Cell Signaling) and Alexa Fluor^®^ 594 goat anti-rabbit secondary antibodies (Invitrogen) as described [26]. Cell images were captured with DMi8b fluorescent microscope (Leica).

### 2.5 Telomere Quantitative Fluorescence in Situ Hybridization (Q-FISH)

Q-FISH was performed as we previously described [28]. Briefly, all cells were maintained in complete medium, treated with 150 μM H_2_O_2_ for 1 hour or left untreated. Then, cells were incubated in medium containing 0.1 μg/ml colcemid (Millipore Sigma) for 4 hours to arrest the cells in metaphase. After adding hypotonic 0.075 M KCl buffer, the cells were fixed in cold methanol/acetic acid (3:1) and kept over-night at 4° C. Metaphase spreads were made and telomere FISH was performed by using Alexa546-conjugated Telomere DNA probe (TTAGGG)x3 (IDT). Chromosomes were counterstained with DAPI. For quantitative assessment of telomere length, digital images of chromosomes in metaphase and telomeres were captured by Nikon CSU-W1 Spinning Disk Confocal microscope, followed by quantitation of telomere size and visualization of telomere fluorescence intensity by using the Telometer plugin (available at http://demarzolab.pathology.jhmi.edu/telometer/index.html) for FIJI software [29]. Additionally, the statical analyses of the average telomere intensities was performed by two-way ANOVAs followed by Fisher’s LSD separate post-hoc comparisons.

### 2.6 Co-immunohistochemistry with Telomere FISH

Following treatment with H_2_O_2_ for one hour and recovery for 4 hours, cells were collected for immunostaining combined with telomere fluorescence in situ hybridization (Immuno-T-FISH) as previously described [25]. High-quality metaphase spreads were prepared and stained as previously described [28]. Briefly, slides were dehydrated with increasing ethanol concentrations, and incubated for 5 min at 87°C with Cy3-conjugated PNA probe (TTAGGG)x3 (Agilent technology Inc., TX), according to the manufacturer’s protocol. Slides were then allowed to anneal at room temperature for 1 hour. Following washes [two washes in wash solution 1 (containing formamide hybridization buffer) for 30 minutes on a shaker, and then followed by 3 washes for 5 minutes with wash solution 2], the primary antibodies, rabbit anti-SIRT6 (1:1000) (Abcam 62739), were diluted in block solution and incubated overnight at 4°C. Slides were then washed and incubated for 1 hour at room temperature with secondary antibodies Alexa 488 Donkey anti rabbit (1:400) (Invitrogen), diluted in block solution. Cells were then counterstained with DAPI and mounted with coverslips. Metaphases were visualized by Nikon CSU-W1 Spinning Disk Confocal microscope. Co-localization study was performed by the JACop plugin [30] for ImageJ software [31]. Colocalization of SIRT6 with telomeres was calculated based on centers of mass-particles coincidence. Results are shown as average ± S.E.M. Data were analyzed by one-way ANOVAs, followed by Tukey’s multiple comparison post-hoc tests.

### 2.7 Southern Blot Analysis

Genomic DNA (5 μg) was purified and treated with restriction enzymes, Rsal and Hinfl at 37°C for one hour following manufacturer’s instructions (New England Biolabs). Digested DNA was separated by 0.7% agarose gel electrophoresis and transferred to a Nylon membrane. Membrane was hybridized with biotinylated Telo-C-probe overnight and then incubated with Streptavidin-HRP for an hour and detected by chemiluminescence detection reagents following manufacturer’s instructions (Thermo Fisher Scientific, Catalog Number: 89880). After treatment with ECL substrate for 5 min, the membrane was exposed to X-ray film.

## 3. Results

### 3.1 hMYH Knockout Human Cells are More Sensitive to Oxidative Stress than Control Cells

**Figure 1:**
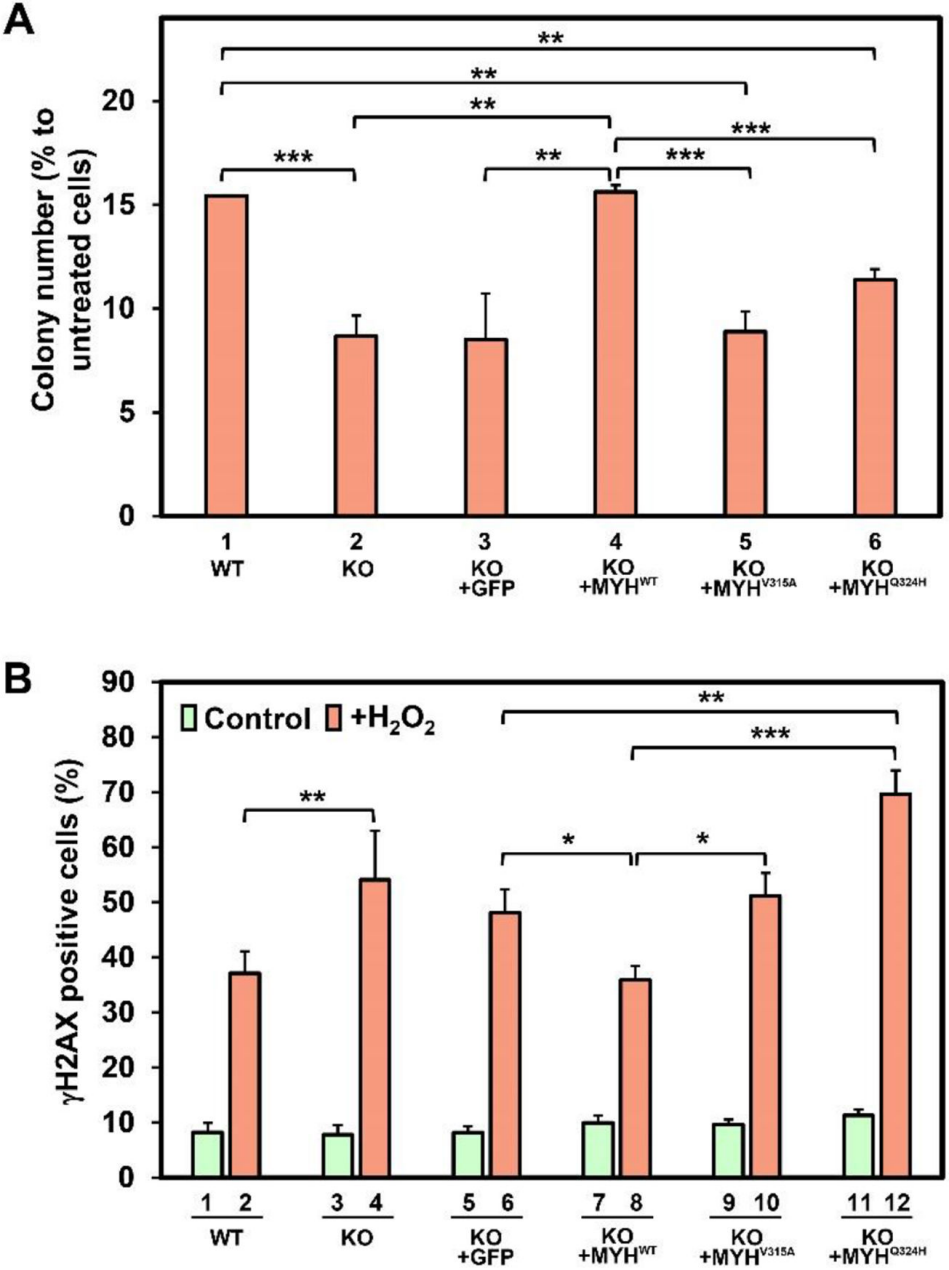
Human *MYH* knockout cells have poor survival rate and higher levels of strand breaks than control cells after H_2_O_2_ treatment; these phenotypes can be complemented by expressing wild type but not mutant hMYH proteins. (A) hMYH-knockout (KO) HEK-293T cells have poor survival rate in comparison to control cells after H_2_O_2_ treatment. GFP (KO+GFP), GFP-MYH^WT^(KO+MYH^WT^), GFP-MYH^V315A^(KO+ MYH^V315A^), and GFP-MYH^Q324H^ (KO+ MYH^Q324H^) were stably expressed in *hMYH* KO cells. Plated cells were treated with 150 μM H_2_O_2_ for 1 hour or left untreated (control), then the plates were incubated for 10 days, and colony formation was analyzed from three experiments. The percentage (%) was calculated from the ratios of H_2_O_2_ treated over untreated samples. (B) hMYH-knockout HEK-293T cells have higher levels of γH2AX than control cells after H_2_O_2_ treatment. Cells were treated with 150 μM H_2_O_2_ for 1 hour or left untreated (control), recovered for 2 hours, and immunofluorescence stained with γH2AX antibody. HEK-293T and KO represent HEK-293T *hMYH*^+/+^ and *hMYH*^−/−^ cells, respectively. Green and orange bars indicate with and without treatment with 150 μM H_2_O_2_ for 1 hour. Representative images are presented in Figure S1. The percentage of γH2AX positive cells was scored from three experiments. The error bars reported are the standard deviations of the averages and *P*-value was calculated using ANOVA followed by separate post hocs analysis. *, **, and *** represent *P*<0.1, *P*<0.05, and *P*<0.01, respectively.

DNA damage caused by H_2_O_2_ treatment includes 8-oxoG [32-34] and DNA strand breaks [35]. We have reported that human HeLa cells with 70%-*hMYH* knockdown (KD) are more sensitive to oxidative stress triggered by H_2_O_2_ than the control *hMYH*^+/+^ cells [27]. We have further shown that mouse Myh (mMyh) foci are induced on telomeres by oxidative stress [25, 26]. Xie *et al*., reported that cells deficient in mMyh and mOgg1 are sensitive to H_2_O_2_ [36]. However, Oka *et al*., have shown that *Myh* knockdown mouse cells are more resistant to H_2_O_2_ [37]. To examine the phenotypes of cells with total deficiency of hMYH, we knocked out (KO) the *hMYH* gene in human HEK-293T cells, resulting in nondetectable hMYH protein [26]. Then, we compared their sensitivity to oxidative stress with the control *hMYH*^+/+^ cells. Cells were treated with medium containing H_2_O_2_, as it has been shown that H_2_O_2_ induces 8-oxoG formation [32, 33]. We found a significant decrease in colony formation in *hMYH* KO HEK-293T as compared to control cells treated with 150 μM of H_2_O_2_ (Figure 1A, compare columns 1 and 2). Thus, hMYH activity is critical to minimize cell death caused by oxidative DNA damage. This is consistent with our previous findings that *hMYH* KO HEK-293T and *hMYH* KD HeLa cells contain higher levels of 8-oxoG and apoptotic cells following peroxide treatment [26, 27].

### 3.2 hMYH Knockout Human Cells Contain Higher Levels of Strand Breaks than Control Cells after H_2_O_2_ Treatment

Our previous results demonstrate that hMYH has a protective role in preventing 8-oxoG accumulation and cell apoptosis following oxidative stress [26, 27]. However, Oka *et al*. [38, 39] showed that Myh promotes DNA strand breaks and induces cell death. To study the molecular mechanism underlying the hMYH-dependent apoptosis, we compared the levels of phosphorylated H2AX (γH2AX) foci, an indicator of DNA strand breaks, in *hMYH* KO and control cells. As shown in Figure 1B, the levels of γH2AX were low in all untreated cells. When control *hMYH*^+/+^ HEK-293T cells were treated with H_2_O_2_, 37% of the cells contained γH2AX foci (Figure 1B, 2nd column; Figure S1). In contrast, 55% of *hMYH* KO cells contained γH2AX after H_2_O_2_ treatment (Figure 1B, 4th column; Figure S1) (*P* = 0.05). Thus, *hMYH* KO triggers DNA strand breaks under oxidative stress.

### 3.3 hMYH Knockout Human Cells Contain Higher Levels of SIRT6 Foci than Control Cells after H_2_O_2_ Treatment

**Figure 2:**
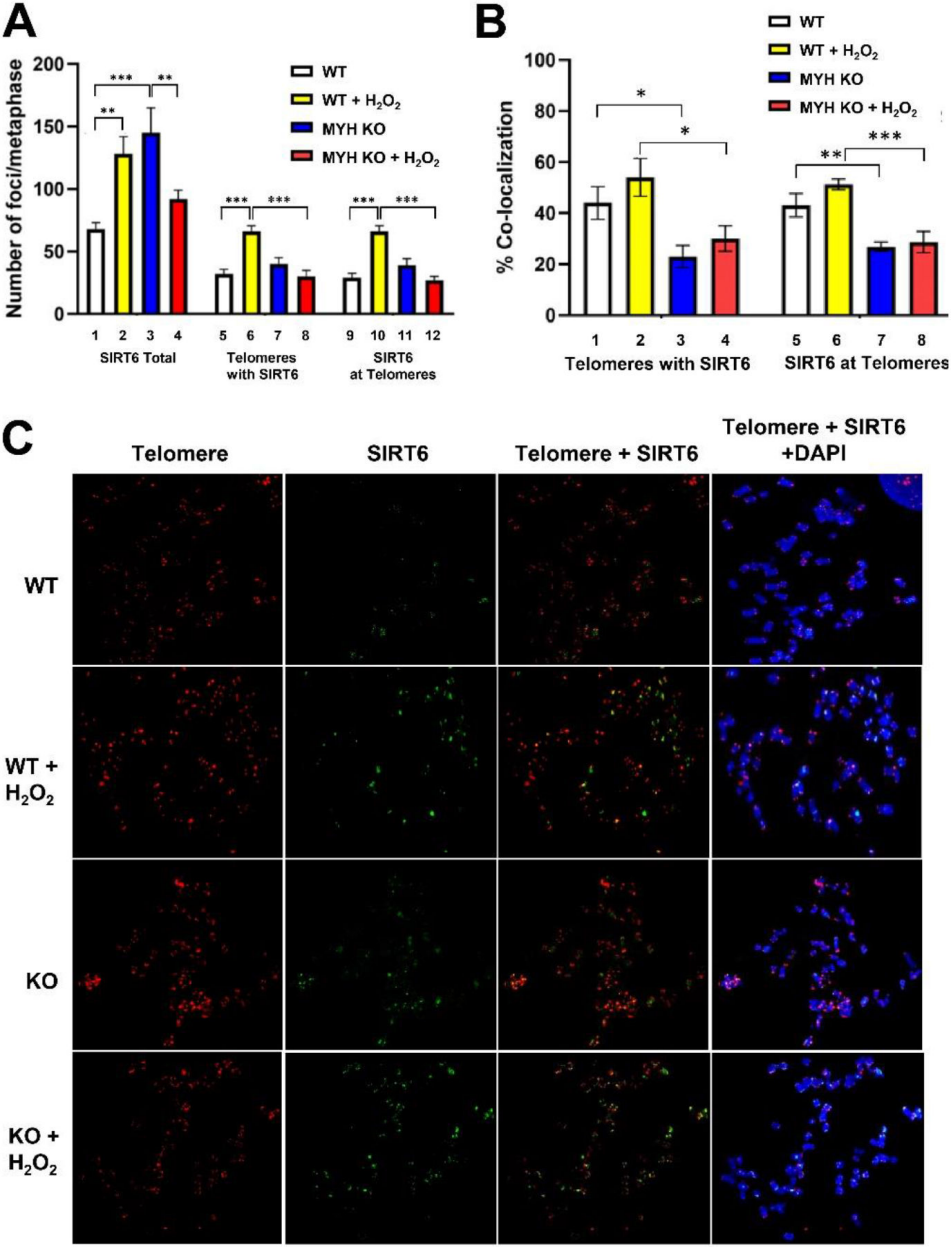
Co-localization analyses of SIRT6 with the telomeres in MYH knockout and control cells. After being treated with H_2_O_2_ for 1 hour and recovered for 4 hours, cells were collected for immuno-telomere FISH. (A), Numbers of SIRT6 foci (green, immunostaining) or telomere signals (red, Alexa 546-conjugated telomere DNA probe) in metaphase spreads (n = 10 per group) were scored by co-localization analyses. Columns 1-4, the total number of SIRT6 foci per metaphase; columns 5-8, the average number of telomere signals which are colocalized with SIRT6 foci; columns 9-12, the average number of SIRT6 foci which are colocalized with telomeres. (B) Percentages of colocalization of telomere and SIRT6 were calculated from (A). Columns 1-4, the percentage of telomere signals with SIRT6 foci and columns 5-8, the percentage of SIRT6 foci at telomere. WT and KO represent HEK-293T *hMYH*^+/+^ and *hMYH*^−/−^ cells, respectively. (C) Representative images showing telomere signal and SIRT6 foci that were merged with DAPI stain in the right panels. Data was analyzed by one-way ANOVA, followed by multiple separate Fisher LSD post-hoc tests. *, **, and *** represent *P*<0.1, *P*<0.05, and *P*<0.01, respectively.

**Figure 3:**
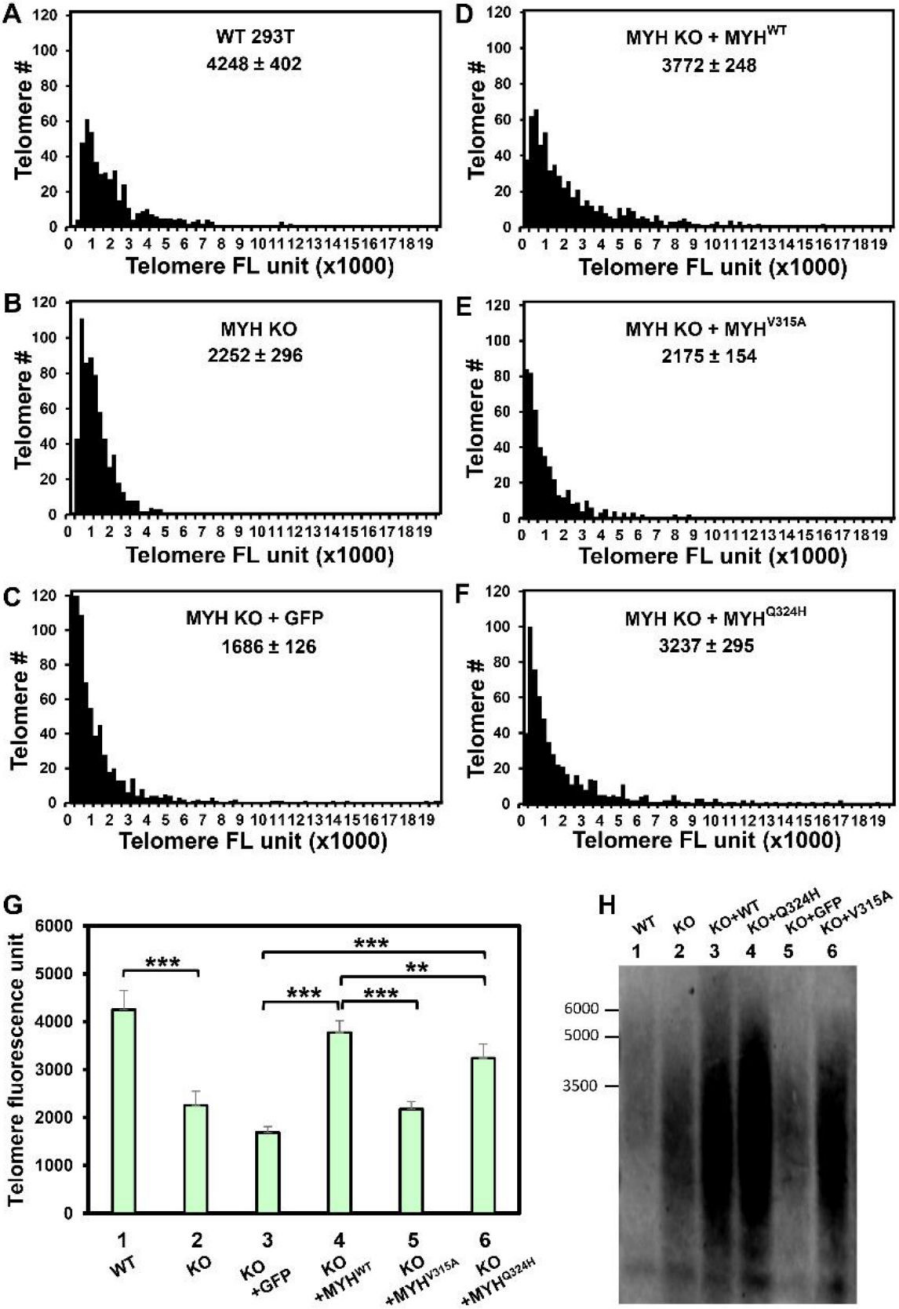
Telomere shortening is induced by hMYH deficiency and is rescued by expression of wild-type hMYH, but not mutant hMYH proteins. Cells were maintained in medium containing 0.1 μg/ml colcemid (Invitrogen) for 4 hours to arrest the cells in metaphase. (A)-(G) were derived from Q-FISH performed by an Alexa 546-conjugated DNA probe as in the Materials and Methods section. (A)-(F), Distribution diagrams of relative telomere length of each group (results of pooled metaphase nuclei, totaling >1000 telomeres). 293T and KO represent HEK-293T *hMYH*^+/+^ and hMYH^−/−^ cells, respectively. GFP-MYH^WT^ (KO+ MYH^WT^), GFP-MYH^V315A^ (KO+V315A), and GFP-MYH^Q324H^ (KO+ MYH^Q324H^) were stably expressed in *hMYH* KO cells. Telomere FL units = telomere fluorescence units. The mean and standard error of the mean (SEM) are indicated. (G) Quantification of average telomeric signal intensities of H_2_O_2_-treated cells by Telometer. The cumulative distribution plots derived from the histograms in (A)-(F) were shown in Figure S2. ** and *** represent *P*<10^−4^ and *P*<10^−5^, respectively. (H) Telomere length was determined by Southern blot analysis. DNA isolated from *MYH*^+/+^ control, *MYH*^−/−^, and *MYH*^−/−^ cells expressing different MYH proteins were digested with restriction enzymes, separated on agarose gel, transferred to membrane, and hybridized with telomere PNA probe as described in the Materials and Methods section. The data validate the effect of MYH knockout on telomere length.

The aging regulator SIRT6 is a NAD^+^-dependent histone/protein deacetylase (reviewed in [40]) and has important roles in stress response, DNA repair, telomere integrity, retro-transposition, and metabolic homeostasis [40-46]. We have shown that SIRT6 protein interacts with MYH [25] and is required for the recruitment of MYH to telomeres [26]. To further delineate the MYH-SIRT6 interaction, we compared SIRT6 foci formation at the global genome and their co-localization with telomeres in the control and *hMYH* KO HEK-293T cells by performing co-immunohistochemistry with Telomere FISH. At the global genome level, there were low levels of SIRT6 foci in untreated control cells, while the levels of SIRT6 foci increased in H_2_O_2_-treated control cells and untreated *hMYH* KO HEK-293T cells (Figure 2A, columns 1-3; Figure 2C, panels 1-3). However, H_2_O_2_ treatment did not increase the levels of SIRT6 foci in *hMYH* KO HEK-293T cells (Figure 2A, compare columns 3 and 4; Figure 2C, panels 3 and 4). The co-localization of SIRT6 foci with telomeres is presented as the numbers of telomeres colocalized with SIRT6 foci and the numbers of SIRT6 foci colocalized with telomeres. SIRT6 foci enrichment was only observed in H_2_O_2_-treated *hMYH*^+/+^ control cells (Figure 2A, columns 5-12). However, the percentages of co-localization in H_2_O_2_-treated control cells remained the same as untreated control cells (Figure 3B, compare column 1 to 2 and column 5 to 6). Interestingly, in untreated *hMYH* KO HEK-293T cells, although the number of SIRT6 foci had increased (Figure 2A, compare columns 1 and 3), co-localization of SIRT6 foci with telomeres did not increase (Figure 2A, compare column 5 to 7 and column 9 to 11). The percentages of co-localization decreased in *hMYH* KO HEK-293T cells as compared to the control cells in the absence or presence of H_2_O_2_ (Figure 2B, compare columns 1 and 2 to columns 3 and 4, compare columns 5 and 6 to columns 7 and 8). Thus, MYH deficiency promotes SIRT6 foci formation mainly at non-telomere genomic regions.

### 3.4 hMYH Knockout Cells Contain Shorter Telomeres and Higher Levels of Telomeric Aberrations than Control Cells after H_2_O_2_ Treatment

It has been shown that oxidative stress to *Myh Ogg1* double KO (but not single KO) mouse embryonic fibroblast cells induces multinucleation accompanied by centrosome amplification and multipolar spindle formation [36]. However, Baquero *et al*. [47] have found that specific inhibition of hOGG1 leads to an accumulation of oxidized bases, that correlates with telomere losses and micronuclei formation. Moreover, *Fouquerel et al*., have shown that hOGG1-deficient cells have shorter telomeres and telomere losses [22]. Because MYH DNA glycosylase is associated with telomeres [25, 26] and plays a more important role than OGG1 in tumorigenesis [17, 48], we examined the contribution of hMYH on telomere integrity in human cells. First, we compared the telomere length in the control and *hMYH* KO cells with and without oxidative stress. Telomere Q-FISH analyses indicated that the average telomere lengths of *MYH*^−/−^ was significantly shorter by 0.53-fold compared to *MYH*^+/+^ control without H_2_O_2_ treatment, (Figure 3A and 3B). The telomere shortening could be easily observed in the cumulative distribution plots derived from the histograms as shown in Figure S2A. Thus, *MYH* KO cells had shorter telomeres than control cells even without H_2_O_2_ treatment (Figure 3G, compare columns 1 and 2). Previous reports have shown OGG1 is important for telomere integrity after long term recovery from repeated oxidative damage to telomeres [22]. To find the immediate effect of acute H_2_O_2_ treatment on the telomere length of *hMYH* KO cells, cells were collected 4 hours after treatment. However, we found that acute H_2_O_2_ treatment did not significantly induce telomere shortening in both *MYH*^+/+^ and *MYH*^−/−^ cells (data not shown).

To validate the effect of MYH knockout on telomere length, we further performed Southern blot analysis for *MYH*^+/+^ control and *MYH*^−/−^ cells (Figure 3H). Our results confirm a substantial telomere shortening in *MYH*^−/−^ cells compared to *MYH*^+/+^ cells. Consistent with the Q-FISH data, the telomere length was very heterogenous. Our findings demonstrate that MYH is required to maintain telomere length.

**Figure 4:**
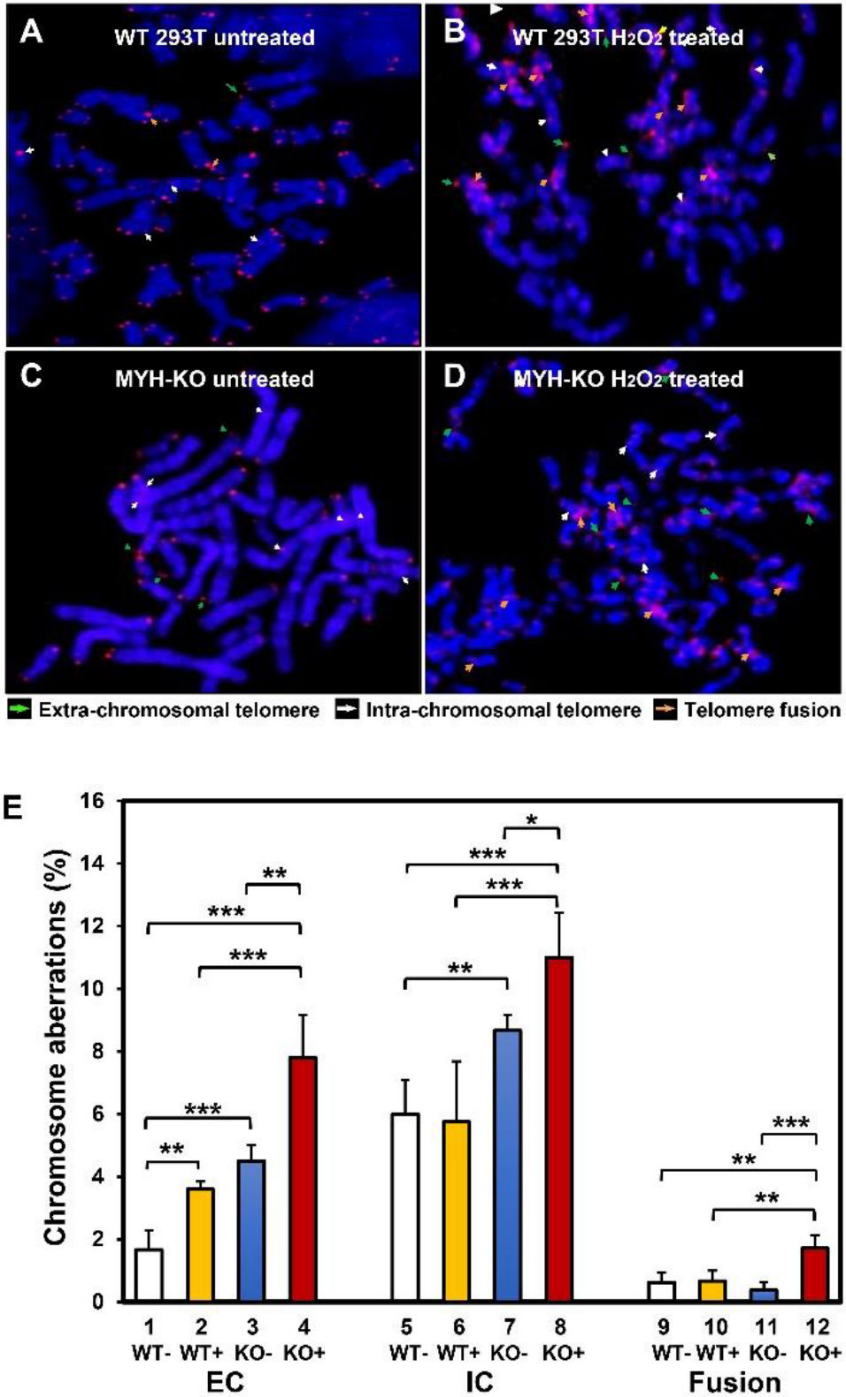
MYH is important for telomere stability. (A-D) Representative images of untreated and H_2_O_2_ treated WT HEK-293T and MYH KO cells. Telomeres were visualized by a PNA probe (red, Cy3-conjugated PNA probe) in metaphase spreads. Chromosomes were stained with DAPI (blue). Arrowheads indicate telomere aberration (green, extrachromosomal telomeres; white, intrachromosomal telomeres; orange, telomere fusions). (E) Telomere MYH KO leads to increase in telomere extra-chromosomal (EC) telomere as well as intra-chromosomal (IC) telomeres even without oxidative damage. Telomere fusion was not significantly increased. H_2_O_2_ treatment significantly increase telomere fusion in *hMYH* KO but not in the control HEK-293T WT cells. More than 1000 chromosomes per cell type were analyzed. WT and KO represent HEK-293T *hMYH*^+/+^ and *hMYH*^−/−^ cells, respectively. (+) and (−) indicate with and without treatment with 150 μM H_2_O_2_for 1 hour. *, **, and *** represent *P*<0.1, *P*<0.05, and *P*<0.01, respectively.

Next, we dissected any of the telomeric aberrations in control and *hMYH* KO cells with and without oxidative stress (Figure 4). The following telomere defects were quantified: telomere fusion, telomere fragmentation, extrachromosomal telomeres, intrachromosomal telomeres and telomere doublets. Data are shown for significantly affected telomere aberrations (per more than 1000 chromosomes) (Figure 4). Representative images of telomere FISH are shown in Figures 4A-4D. *hMYH*-KO HEK-293T cells had increased extra-chromosomal and intra-chromosomal telomeres in comparison to the control cells even without H_2_O_2_ treatment (Figures 4E, columns 1, 3, 5, and 7). H_2_O_2_ treatment increased only the frequency of extra-chromosomal telomere repeat DNA in control HEK-293T cells (Figures 4E, compare columns 1 and 2). Remarkably, the frequencies of extra-chromosomal telomeres, intra-chromosomal telomeres, and telomere fusions were further increased in *hMYH* KO cells after H_2_O_2_ treatment (Figures 4E, compare column 4 with column 3, compare column 8 with column 7, and compare column 12 with column 11). *hMYH*-KO HEK-293T cells had increased extra-chromosomal telomeres, intra-chromosomal telomeres, and telomere fusions in comparison to the control cells after H_2_O_2_ treatment (Figures 4E, columns 2, 4, 6, 8, 10, and 12). However, the frequencies of fragmented telomeres and telomere doublets were not altered by peroxide treatment and MYH deletion (data not shown). Therefore, our results indicate that MYH deficiency and oxidative stress can contribute to telomere instability.

### 3.5 The Phenotypes of hMYH KO Cells are Corrected by Expressing Wild-type hMYH, but not by Expressing hMYH^V315A^ and hMYH^Q324H^ Mutants

Next, we examined whether the phenotypes of *hMYH* KO cells can be complemented by the expression of wild-type or mutant MYH proteins. We stably expressed GFP-MYH^WT^, GFP-MYH^V315A^, or GFP-MYH^Q324H^ in *hMYH* KO HEK-293T cells. GFP-tagged hMYH proteins were equally expressed as indicated by Western blotting (data not shown). V315 and Q324 are located within the interdomain connector (IDC, residues 295-350) of hMYH that serves as a scaffold for interactions with Hus1, SIRT6, and APE1 (second enzyme in BER pathway) [25, 49, 50]. MYH^V315A^ is defective in interactions with Hus1 and SIRT6, while MYH^Q324H^ is defective in Hus1 interaction, but not with SIRT6 [17, 25, 46]. Our results demonstrate that expression of wild-type hMYH in H_2_O_2_-treated *hMYH* KO cells restored the H_2_O_2_ resistance back to the wild-type levels (Figure 1A, compare columns 1 and 4). However, expression of hMYH^V315A^ or hMYH^Q324H^ could not completely complement the defects in *hMYH* KO cells (Figure 1A, compare columns 5 and 6 to column 4). hMYH^V315A^ was more defective than hMYH^Q324H^ in restoring the colony formation of *hMYH* KO cells.

Expression of wild-type hMYH in H_2_O_2_-treated *hMYH* KO cells also reduced the levels of γH2AX back to the wild-type levels (Figure 1B, compare columns 2 and 8). However, expression of hMYH^V315A^or hMYH^Q324H^ could not reduce the levels of γH2AX in *hMYH* KO cells (Figure 1B, compare columns 10 and 12 to column 8). The level of γH2AX in *hMYH* KO cells expressing hMYH^Q324H^ was higher than that of empty vector-transfected *hMYH* KO cells (Figure 1B, compare columns 6 and 12). Thus, interrupting the interactions of MYH with its partners can increase cellular sensitivity to H_2_O_2_ and elevate cellular DNA strand breaks.

Finally, we examined whether expression of wild-type hMYH in *hMYH* KO cells could restore the telomere length back to the wild-type levels. The telomere length as measured by the Q-FISH of untreated *hMYH* KO cells was rescued with wild-type hMYH expression and was similar to that of *hMYH*^+/+^ cells. Interestingly, telomere length was substantially longer than that of KO cells transfected with GFP vector (Figure 3D; Figure 3G, compare column 4 with columns 1 and 3; as well as Figure S2B). However, expression of hMYH^V315A^ in *hMYH* KO cells could not lengthen the telomeres to control level (Figure 3E; Figure 3G, compare column 5 to columns 3 and 4; as well as Figure S2C and S2E). Expression of hMYH^Q324H^ in *hMYH* KO cells could only partially restore the telomere length [(Figure 3F; Figure 3G, compare column 6 to columns 3 and 4; as well (Figure S2D and S2F)]. Consistent with the Q-FISH data, Southern blot results show that while telomeres are extended by the expression of MYH^WT^and MYH^Q324H^, length is only partially rescued by MYH^V315^, yet some of the telomere lengths were more heterogenous compared to the control cells. Thus, interrupting the interactions of MYH with its partners can affect its role in maintaining telomere length.

## 4. Discussion

**Figure 5:**
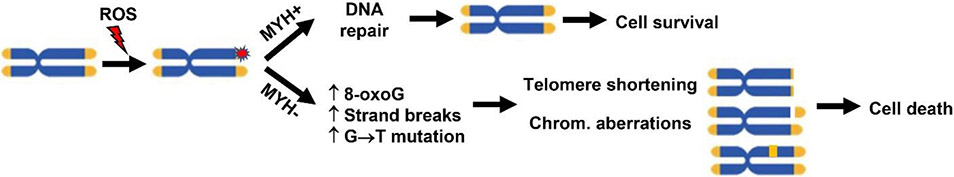
A model depicting MYH functions at telomeres controlling telomere stability and cell viability. MYH has a protective role in cell survival under oxidative stress by reducing the levels of 8-oxoG, DNA strand breaks, and G:C to T:A mutations. Defect in MYH can cause telomere shortening and telomere aberrations, leading to cell death.

MYH plays multifaceted roles in maintaining genomic stability to prevent mutagenesis and tumorigenesis. MYH acts on transcriptionally active genomes and telomeres to reduce G:C to T:A mutations caused by oxidatively damaged 8-oxoG [25, 26]. Besides its function in mutation avoidance, MYH also has other functions such as checkpoint activation and apoptosis avoidance upon DNA damage [17, 27, 48, 49]. Our current results show that *hMYH* knockout human HEK-293T cells are more sensitive to oxidative stress, have shorter telomeres, and increase SIRT6 foci at the global genome. In addition, *hMYH* knockout cells contain higher levels of DNA strand breaks and telomeric aberrations than control cells under oxidative stress. We have shown that oxidatively stressed *hMYH* KO HEK-293T cells contain higher levels of 8-oxoG and are prone to induce apoptosis [26]. These collective properties of *MYH* KO human cells support a protective role of MYH in cell survival under oxidative stress (Figure 5). Although HEK-293T cells express SV40 T antigen which may inactivate p53 [51], multiple lines of evidence suggest that the MYH-mediated DNA damage repair pathway in HEK293T cells, is similar to that in normal human or mouse cells. First, we have reported that *hMYH* KD HeLa human cells are more sensitive to oxidative stress with increased levels of apoptosis and 8-oxoG lesions [27]. Second, the findings of Molatore [52] *et al. and* Turco *et al*. [53] show, that *mMyh* knockout mouse cells contain higher 8-oxoG and are more sensitive to oxidative stress than the *mMyh* positive cells. However, Oka *et al*. [26, 27] observed that knockdown of *mMyh* reduces mouse cell death. The differences between the roles of the human and the mouse MYH remains to be elucidated.

We found that *hMYH* KO cells contain higher levels of γH2AX than control cells after H_2_O_2_ treatment. Phosphorylated H2AX (γH2AX) has been used as an indicator for double strand breaks [54], however, the main fraction of γH2AX induced by oxidative stress does not appear to depend on double strand breaks, and is mediated by TopBP1-dependent ATR kinases [55]. It has been shown that MYH plays an important role in ATR signaling by interacting with the checkpoint clamp Rad9/Rad1/Hus1 (the 9-1-1 complex) and TopBP1 [56]. After adenine excision by MYH, the AP site of DNA product is converted to single-stranded breaks by APE1 endonuclease. We hypothesize that strand breaks do not accumulate in MYH proficient cells to avoid apoptosis under oxidative stress because MYH and the 9-1-1 complex stimulate APE1 activity [49, 50, 57-59]. The 9-1-1 complex has been proposed to coordinate the BER process by a “passing the baton” mechanism to avoid the accumulation of BER intermediates (reviewed in [60, 61]). Accordingly, the product of MYH is safely passed to APE1 to drive the BER pathway to completion. Our result that γH2AX level is increased in *hMYH* KO cells expressing hMYH^Q324H^ mutant is consistent with this “passing the baton” mechanism. Alternately, the interaction of MYH with SIRT6 protein deacetylase [25] may stimulate the activity of PARP-1 [62], which binds to AP sites and single-stranded breaks [63], leading to enhanced BER [38]. Our result that γH2AX level is increased in *hMYH* KO cells expressing hMYH^V315A^ mutant suggests that MYH interactions with 9-1-1 and SIRT6 are critical to minimize strand break production during base excision repair. Because strand breaks at telomeric DNA are poorly repaired [21, 61], their protection and avoidance by MYH and associated factors may abate telomere shortening and aberrations.

We show, for the first time, that *hMYH* KO HEK-293T cells contained not only shorter telomeres, but also higher frequencies of chromosomal aberrations as compared to the control cells. These increased telomere defects can be attributed to increased oxidative damage and decreased cell viability in *hMYH* KO cells. Our findings that *hMYH* KO cells contain high levels of γH2AX and 8-oxoG [26] support previous notions that single-stranded breaks and 8-oxoG are the major causes of telomere shortening and chromosomal aberrations [22, 64]. MYH can reduce 8-oxoG accumulation and 8-oxoG-induced G:C to T:A mutations [15, 25, 26] which can alter telomere structure and binding of telomere-associated factors [65]. Sun *et al*., [23] have shown that chromatid telomere loss and telomere fusions are likely the signature chromosomal aberrations at oxidatively damaged telomeres. We did not assess telomere loss, as some of the telomeres of HEK-293T cells were below the detection level. Our results indicate a significant increase of extra-chromosomal and intra-chromosomal telomeres in *MYH* KO cells even without external oxidative stress. H_2_O_2_ treatment increases the frequencies of extra-chromosomal telomeres, intra-chromosomal telomeres, and telomere fusions in *MYH* KO cells, but only an increase in the frequency of extra-chromosomal telomeres in control HEK-293T cells. It is interesting to note that telomere fusion is only increased in H_2_O_2_-treated cells but not in untreated *MYH* KO cells. Severe oxidative stress can produce clustered DNA damages which may generate double-stranded breaks in addition to single-stranded breaks. Because MYH does not directly remove 8-oxo-G, we have hypothesized that MYH-directed BER pathway may convert A/G° to C/G° which is then repaired by OGG1 or by other repair pathways to promote cell survival [26]. In addition, tight binding of MYH with G° mispaired with T, G, and abasic sites, may block adverse OGG1 glycosylase activity from generating strand breaks [66]. Thus, in *hMYH* KO and hOGG1 positive human cells, uncontrolled hOGG1 excision activity on G°-containing strands of DNA with G°/AP, G°/C, G°/T, and G°/G mismatches may trigger telomere instability and cell death.

SIRT6 plays a significant role in maintaining chromosomal stability [67]. SIRT6 is one of the enzymes most rapidly recruited at sites of DNA damage [26, 38] and participates in BER [62, 67-69]. Our previous publication provides a direct functional role of SIRT6 in BER through interaction with MYH, APE1, and the 9-1-1 complex [25]. In this report, we compare SIRT6 foci formation and co-localization with telomeres in both *hMYH*^+/+^ and *hMYH*^−/−^ cells. At the global genome level, the levels of SIRT6 foci increased in the H_2_O_2_-treated control cells and untreated *MYH* KO cells, but only slightly increased in H_2_O_2_-treated *MYH* KO cells. The levels of SIRT6 foci increased at both the global genome and telomeric regions in H_2_O_2_-treated *MYH*^+/+^ cells (Figure 2A, columns 2, 6, and 10). However, in untreated *MYH* KO HEK-293T cells, SIRT6 foci only increase at the global genome but not at telomeric regions (Figure 2A, columns 3, 7, and 11). Although telomeric sequences only represent a small proportion of the human genome, over 40% of SIRT6 foci are colocalized with telomeres in *hMYH*^+/+^ cells (Figure 2B). The percentages of co-localization of SIRT6 foci with telomeres were reduced to about 25% in *hMYH* KO cells. We suggest that SIRT6 responds to oxidative damage at heterochromatin in *hMYH*^−/−^ cells because hMYH acts on euchromatin and telomeres [25, 26]. Our results indicate that SIRT6 interaction with MYH is critical for its recruitment with the telomere. Moreover, expression of WT, but not GFP-hMYH^V315A^, in *hMYH* KO cells restores the H_2_O_2_ resistance, reduces strand breaks, and increases telomere length back to the wild-type levels. Our previous findings showed that the association of hMYH with damaged telomeres is substantially attenuated in *Sirt6* KO mouse cells, but SIRT6 recruitment to damaged telomeres is independent of MYH [26]. The discrepancy of these findings may be due to oxidative damage to the entire genome in human cells in this report and local damage on telomeres in mouse cells in the paper of Tan *et al*. [26]. Our new data suggest a mutual relationship between MYH and SIRT6. An initial MYH recruitment by SIRT6 to telomeres is necessary to facilitate the SIRT6-BER protein complex formation and to maintain telomere stability. However, when cells are severely damaged, clustered oxidative damage accompanied with SIRT6 foci formation in *MYH* KO cells may result in chromosome aberrations and cell death (Figure 5).

## 5. Conclusions

In this paper, we show that *hMYH* knockout human HEK-293T cells are more sensitive to oxidative stress, have shorter telomeres, and contain higher levels of DNA strand breaks, SIRT6 foci, and telomeric aberrations in comparison to the control cells while under oxidative stress. Interrupting the MYH interactions with SIRT6 aging regulator and 9-1-1 checkpoint clamp can affect MYH functions on cell survival, strand break formation, and telomere length maintenance. Together with previous studies, our results support that MYH is a key factor to reduce the levels of 8-oxoG and strand breaks, in turn, maintaining genomic stability and telomere integrity.

## Supplementary Material

Supplement Figure S1: Representative images of γH2AX staining of H_2_O_2_-treated cells in Figure 1B. Cells as indicated were treated with 150 μM H_2_O_2_ for 1 hour, recovered for 2 hours, and immunofluorescence stained with γH2AX antibody. 293T and KO represent HEK 293T *hMYH*^*+/+*^ and *hMYH*^*−/−*^ cells, respectively. Cells were co-stained with DAPI and cells transfected with GFP plasmid were detected with GFP fluorescence.
Figure S2: The cumulative distribution plots derived from the histograms of (A)-(F) in Figure 3 in the text were shown. Significance between each pair was determined at 95% confidence interval. 293T WT and KO represent HEK 293T *hMYH*^*+/+*^ and *hMYH*^*−/−*^ cells, respectively. GFP (KO-GFP), GFP-MYHWT(KO-WT), GFP-MYHV315A(KO-V315A), and GFP-MYHQ324H (KO-Q324H) were stably expressed in *hMYHKO* cells.
