# Supplementary material for: Mammalian MutY Homolog (MYH or MUTYH) is Critical for Telomere Integrity under Oxidative Stress
Source: OBM Geriat. Author manuscript; Available in PMC 2022 Jul 8. (PMC9267527; doi:10.21926/obm.geriatr.2202196)
Supplement: Supplement — Figure S1: Representative images of γH2AX staining of H2O2-treated cells in Figure 1B. Cells as indicated were treated with 150 μM H2O2 for 1 hour, recovered for 2 hours, and immunofluorescence stained with γH2AX antibody. 293T and KO represent HEK 293T hMYH+/+ and hMYH−/− cells, respectively. Cells were co-stained with DAPI and cells transfected with GFP plasmid were detected with GFP fluorescence. Figure S2: The cumulative distribution plots derived from the histograms of (A)-(F) in Figure 3 in the text were shown. Significance between each pair was determined at 95% confidence interval. 293T WT and KO represent HEK 293T hMYH+/+ and hMYH−/− cells, respectively. GFP (KO-GFP), GFP-MYHWT(KO-WT), GFP-MYHV315A(KO-V315A), and GFP-MYHQ324H (KO-Q324H) were stably expressed in hMYHKO cells. [file NIHMS1818692-supplement-Supplement.pdf]

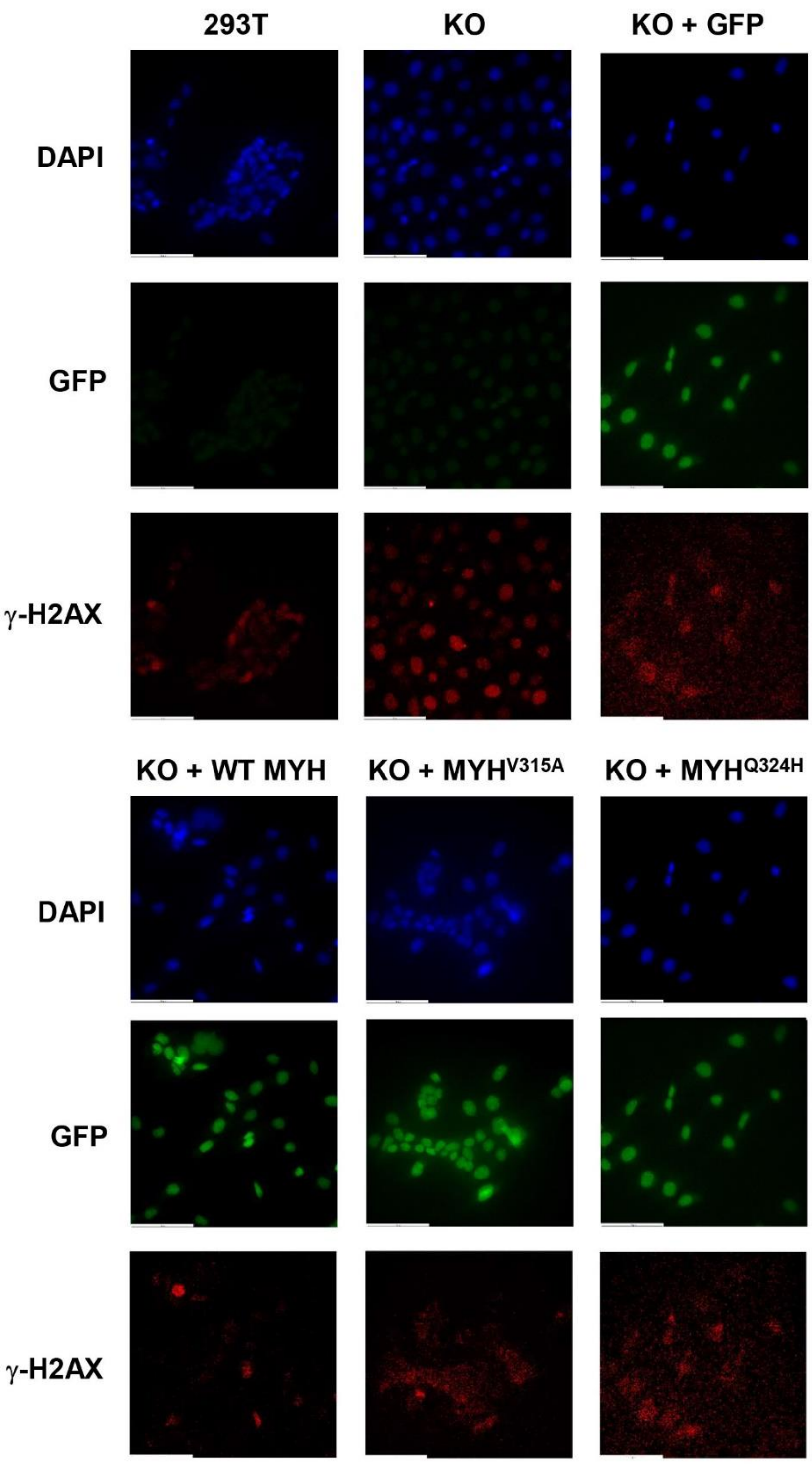

Figure S1 Representative images of γH2AX staining of H_2O_2-treated cells in Figure 1B. Cells as indicated were treated with 150 μM H_2O_2 for 1 hour, recovered for 2 hours, and immunofluorescence stained with γH2AX antibody. 293T and KO represent HEK 293T *hMYH*$^{+/+}$ and *hMYH*$^{-/-}$ cells, respectively. Cells were co-stained with DAPI and cells transfected with GFP plasmid were detected with GFP fluorescence.

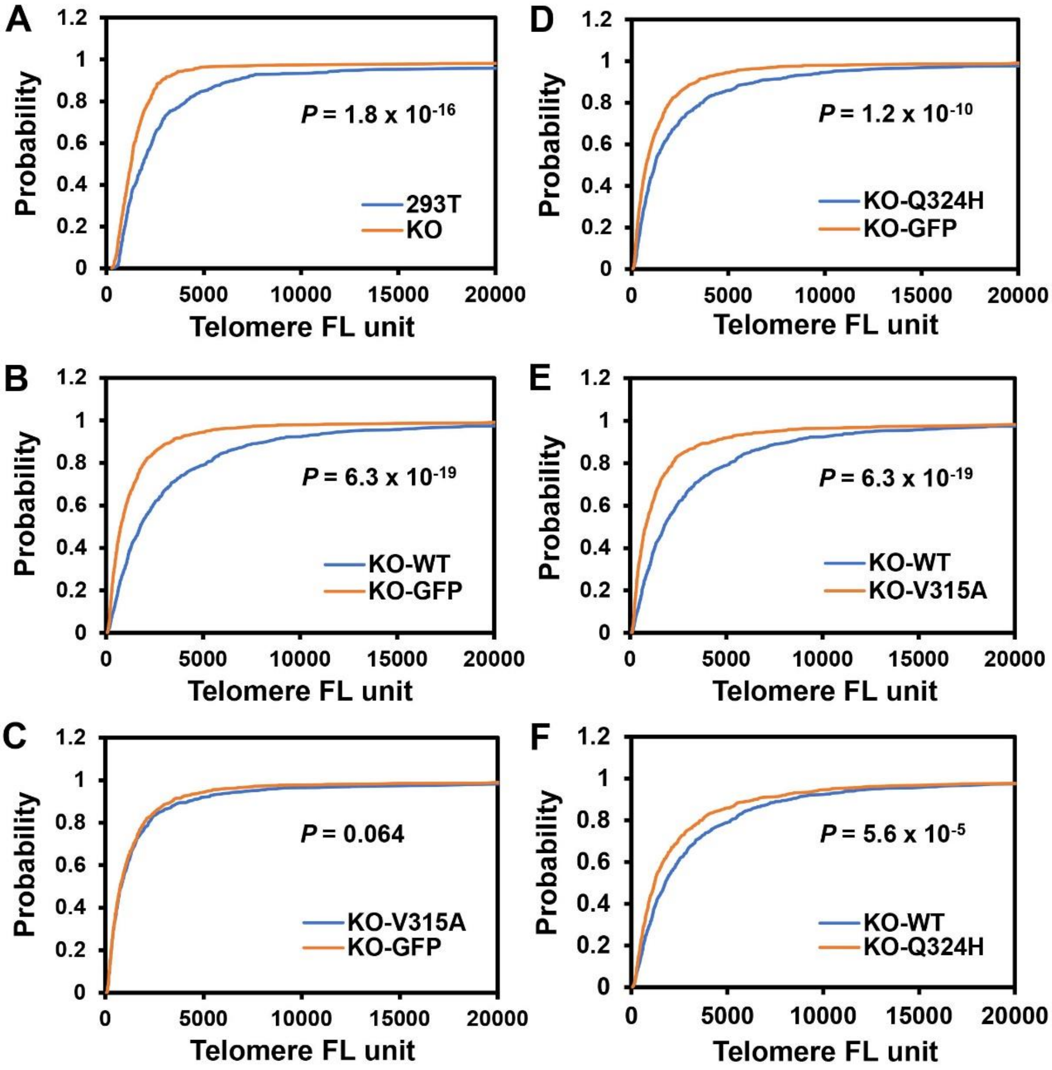

Figure S2 The cumulative distribution plots derived from the histograms of (A)-(F) in Figure 3 in the text were shown. Significance between each pair was determined at 95% confidence interval. 293T WT and KO represent HEK 293T *hMYH*$^{+/+}$ and *hMYH*$^{-/-}$ cells, respectively. GFP (KO-GFP), GFP-MYHWT(KO-WT), GFP-MYHV315A(KO-V315A), and GFP-MYHQ324H (KO-Q324H) were stably expressed in *hMYHKO* cells.
